# Sexually Dimorphic Response of Increasing Dietary Intake of High Amylose Wheat on Metabolic and Reproductive Outcomes in Male and Female Mice

**DOI:** 10.3390/nu12010061

**Published:** 2019-12-25

**Authors:** See Meng Lim, Amanda J. Page, Hui Li, John Carragher, Iain Searle, Sarah Robertson, Beverly Muhlhausler

**Affiliations:** 1 School of Agriculture, Food and Wine, The University of Adelaide, Glen Osmond 5064, Australia; 2 South Australian Health and Medical Research Institute, Adelaide 5000, Australia; 3 Centre for Community Health, Universiti Kebangsaan Malaysia, Kuala Lumpur 50300, Malaysia; 4 Adelaide Medical School, The University of Adelaide, Adelaide 5000, Australia; 5 School of Biological Sciences, The University of Adelaide, Adelaide 5005, Australia; 6 Robinson Research Institute, The University of Adelaide, Adelaide 5000, Australia; 7 Commonwealth Scientific and Industrial Research Organisation, Adelaide 5000, Australia

**Keywords:** high amylose wheat, low glycaemic index, metabolic health, reproductive function

## Abstract

High amylose wheat (HAW) has a higher resistant starch content and lower glycaemic index than standard amylose wheat (SAW), which may be associated with health benefits. This study aimed to determine the effects of replacing SAW with HAW on metabolic and reproductive parameters in male and female mice. Male and female C57BL/6 mice were randomly divided into groups (*n* = 8/group/sex) and fed either a SAW65 (65% SAW w/w; control), HAW35 (35% HAW w/w), HAW50 (50% HAW w/w) or HAW65 (65% HAW w/w) diet for eight weeks. In male but not female, the HAW65 group had a lower abdominal circumference, relative total fat mass, relative gonadal fat mass and plasma leptin concentration compared to the HAW35 group. There were no differences in fasting blood glucose concentrations or plasma concentrations of cholesterol, triglycerides or non-esterified fatty acids between groups in either males or females. The HAW-fed males had a higher testicular weight and HAW-fed females spent less time in diestrus and a longer time in metestrus compared to the SAW-fed mice. Higher dietary intake of HAW appears to reduce abdominal fat deposition compared to the lower level of HAW in a sexually dimorphic manner. The impacts on reproductive parameters in the HAW-fed mice require further investigation.

## 1. Introduction

Whole grain flours are nutritionally superior to refined flours, since they contain the fibre, vitamins, minerals and phytochemicals that are mainly present in the bran and germ and have been reported to contribute to positive health effects [1]. Recent meta-analyses provide evidence of an inverse association between whole grain intake and risk of non-communicable diseases including cardiovascular disease, type 2 diabetes mellitus, certain cancers and obesity [2,3]. Higher whole grain intake has also been reported to improve fertility health parameters, notably increasing the probability of embryo implantation and live birth among women undergoing in vitro fertilisation [4].

Despite the established benefits of whole grains, most people worldwide are still consuming greater quantities of refined grain products compared to whole grains [5]. In a survey of 12,153 Australian adults conducted between 2011 and 2013, more than 70% failed to meet the recommended daily target intake for whole grains of 48 g [6]. Thus, there remains a need to develop strategies for increasing, and maintaining in the long term, whole grain intake at a population level. The most effective solution is likely to be the incorporation of whole grains into existing staple food products, so that consumers are not required to alter dietary habits in order to increase intake. Bread wheat (*Triticum aestivum* L.) is an important staple cereal grain food for humans throughout the world, but especially in Western-style diets. The transition to increased consumption of wheat and wheat-based products in many Asian populations further contributes to the demand for wheat worldwide [7]. Wheat is used in a wide range of food products, including bread, cookies, pastries, breakfast cereal and noodles. Therefore, any improvements in the nutritional properties of wheat has considerable potential to increase the nutritional quality of a large number of end-user products consumed by many people worldwide.

High amylose wheat (HAW) is a novel wheat type with additional nutritional benefits compared with standard wheat products. It contains higher levels of amylose compared to standard wheat [8], with 38% to 85% amylose [9,10] compared to ~28% in standard wheat varieties [11]. The higher amylose content is correlated with increased resistant starch [12], with HAW types being reported to have a resistant starch content as high as 11.2% compared to negligible amounts (<1%) in standard wheat [12]. The increased linear chain structure of amylose confers greater resistance to digestive enzymes, and thus this wheat has a lower glycaemic index [13]. The greater amount of resistant starch in HAW, which is not absorbed in the small intestine, further reduces the availability of carbohydrate absorption.

Lower glycaemic index and high resistant starch diets have been reported to be associated with reduced risk of metabolic diseases, including obesity, type 2 diabetes mellitus and cardiovascular disease [14,15,16]. Consequently, the properties of HAW have led to the suggestion that partial or complete replacement of standard wheat with HAW in staple foods may have beneficial effects on metabolic health, without consumers needing to dramatically change their dietary composition [8]. However, few studies have directly tested the effect of increased HAW consumption on metabolic health outcomes in either humans or experimental animal models. The level of HAW which may be required to improve metabolic health outcomes, and whether the effects of HAW differ between males and females [17], remain also unclear. Therefore, the aim of this study was to evaluate the effect of replacing standard wheat flour in the diet with increasing levels of HAW on growth, food intake, fat mass, nutrient metabolism and plasma metabolic parameters in male and female mice. Given the established association between metabolic and reproductive health [18], we also sought to examine the effect of the HAW diet on some measures of reproductive function in both genders.

## 2. Materials and Methods

### 2.1. Wheat Materials

Commercially available standard amylose wheat (SAW; *Triticum aestivum* L. var. Stylet) and HAW were provided by the Waite Research Institute, The University of Adelaide (Glen Osmond, SA, Australia). The HAW was developed through a normal selective breeding process and has ~46% amylose in its total starch content. The nutrient composition of HAW was analysed by the Australian Export Grains Innovation Centre (North Ryde, NSW, Australia) and is detailed in Table S1 (Supplementary Materials). The dehulled grains of normal bread wheat and HAW were ground to a fine wholegrain flour using a small stone mill (Schnitzer Vario, Offenburg, Germany). The wheat flours were then cooked, in order to reflect how humans typically consume grains. Briefly, each whole wheat flour (HAW, SAW or blend) was mixed with distilled water (1:1.1–1.5 w/v) before being baked at 70 °C and 80% humidity for 1 h, and then cooled overnight at 4 °C to promote retrogradation.

### 2.2. Animals and Dietary Interventions

All animal procedures were approved by the South Australian Health and Medical Research Institute (SAHMRI) Animal Ethics Committee (Project code: SAM294) and were in compliance with the Australian National Health and Medical Research Council’s code for the care and use of animals for scientific purposes (8th edition 2013) and South Australia Animal Welfare Act 1985.

Eight-week-old inbred C57BL/6 male and female mice (*n* = 64) were acquired from the SAHMRI specific pathogen-free and PC2 animal facility breeding colony (Adelaide, SA, Australia) from animals originally obtained from The Jackson Laboratory (Bar Harbor, ME, USA). This strain was chosen because it is one of the most widely used inbred strains of laboratory mouse [19], particularly for metabolic studies [20]. Mice of the same sex were group housed, four to a cage, and maintained within a temperature-controlled environment (22 ± 2 °C) under a 12 h light–dark cycle with *ad libitum* access to food and autoclaved reverse osmosis water throughout the experiment. Mice were randomly assigned to one of four treatment groups (*n* = 8/group/sex) and received one of four diets containing wheat flour (made in-house) for eight weeks. The four diets replaced some of the carbohydrate component of the AIN-93M formulation with SAW and/or HAW—the SAW65 diet contained 65% SAW (w/w); the HAW35 diet contained 35% HAW (w/w) and 30% SAW (w/w); the HAW50 diet contained 50% HAW (w/w) and 15% SAW (w/w); and the HAW65 diet contained 65% HAW (w/w; Table 1). The diets were all vacuum-packed and sent for irradiation at 25 kGy (Steritech, Dandenong, VIC, Australia) prior to storage at −20 °C until use. A sample of each irradiated diet was sent for nutrient analysis by National Measurement Institute (Port Melbourne, VIC, Australia) and Australian Export Grains Innovation Centre (North Ryde, NSW, Australia). The nutrient analysis of each diet is presented in Table 2.

### 2.3. Body Weight and Food Intake

The body weight (BW) of each mouse was measured at weekly intervals throughout the study period. Food intake (g/mouse/day) was determined for the first six weeks of the study by calculating the differences between initial and final weight of the food in the cages (including any spilled food in the cages) over a three- or four-day period and dividing by the number of days and number of mice in each cage. Energy intake (kcal/mouse/day) was calculated based on the energy content of the feed, as determined by nutritional analysis (Table 2).

### 2.4. Metabolic Measurement

On weeks 7 and 8 of the intervention, individual mice were placed into a metabolic monitoring cage (Promethion, Sable Systems International, Las Vegas, NV, USA). After a 24 h acclimatisation, the energy expenditure, oxygen consumption, carbon dioxide production, water intake and physical activity (ambulatory) of each mouse was recorded over a 48 h period. Respiratory exchange quotient (RQ) was calculated as the ratio of carbon dioxide production over oxygen consumption. Data were analysed and presented separately for the two 12 h cycles (12 h light (sleep) phase and 12 h dark (active) phase) and the average (energy expenditure and RQ) or sum (water intake and ambulatory activity) of these two cycles.

### 2.5. Oestrus Cycle Assessment

After three weeks on the respective diets, the oestrus cycle stage of female mice was determined daily, except when mice were housed in metabolic cages. This was achieved by light microscope analysis of cell smears obtained after gently flushing the vaginal opening with saline [21]. The procedure was conducted at the same time of day (10:00 a.m. to 12:00 p.m.) to reduce variability. The stage of the oestrus cycle was identified based on the presence, absence or proportion of leukocytes and cornified epithelial and nucleated epithelial cells [21]. One oestrus cycle was considered as the sequence of proestrus, oestrus, metestrus and diestrus. Data were analysed and presented as the average number and length of oestrous cycles and the number of occurrences of each stage over 24 days from weeks 4 to 7.

### 2.6. Blood and Tissue Collection

After eight weeks on their respective diets, mice were separated into individual cages and fasted overnight, with coprophagy restricted using a raised wire mesh inserted into the bottom of the mouse cages to allow faecal pellets to drop to the cage floor. On the following morning, mice were anaesthetised by isoflurane inhalation and endpoint BW, abdominal circumference and body length (nose to anus) of each mouse were measured. Blood was collected via the abdominal aorta into EDTA tubes, centrifuged (1000× *g*, 15 min, 4 °C) to separate red blood cells and plasma, and the plasma fraction snap frozen in liquid N_2_ and stored at −80 °C for later analysis. Mice were then euthanised by cervical decapitation. Liver, adipose tissues (gonadal, retroperitoneal, mesentery and interscapular depots), kidneys, pancreas, spleen and reproductive organs (testes or uterus and ovaries) were dissected and weighed. All collections were carried out within 1 to 2 h after the beginning of the light phase to minimise possible circadian effects. The majority of female mice were sampled during either the diestrus (72%) or metestrus (19%) stage of the oestrus cycle.

### 2.7. Biochemical Analyses

Glucose was determined using a blood glucose meter (Accu-Chek, Roche, Basel, Switzerland). Plasma concentrations of total cholesterol (Roche Diagnostics Ltd., Rotkreuz, Switzerland), triglycerides (Roche Diagnostics Ltd., Rotkreuz, Switzerland) and non-esterified fatty acids (Wako Pure Chemical Industries, Osaka, Japan) were measured at the Adelaide Research Assay Facility using a COBAS Integra 400 analytical system (Roche Diagnostics Ltd., Rotkreuz, Switzerland). The minimum detection limit of the assay was 0.1 mmol/L for total cholesterol and triglycerides and 0.05 mmol/L for non-esterified fatty acids. The intra-assay coefficients of variation were 4.3% (total cholesterol), 6.1% (triglycerides) and 11.0% (non-esterified fatty acids). The leptin concentration was determined using the Milliplex xMAP Luminex Assay (Merck Millipore, Temecula, CA, USA). The minimum detection limit for leptin was 0.04 ng/mL and the intra-assay coefficient of variation was 8.3%.

### 2.8. Statistical Analysis

Data are presented as mean ± SEM. Data analysis was performed separately on each sex. Two-way ANOVA with Tukey’s post hoc test was used where appropriate to determine significant differences in BW, food intake and energy intake over time. Differences between groups in other measures were determined using one-way ANOVA with Tukey’s post hoc test where appropriate. All statistical analyses were carried out using GraphPad Prism software (version 7.04, San Diego, CA, USA). Significance was considered at *p* < 0.05.

## 3. Results

### 3.1. Food Intake and Energy Intake

**Figure 1:**
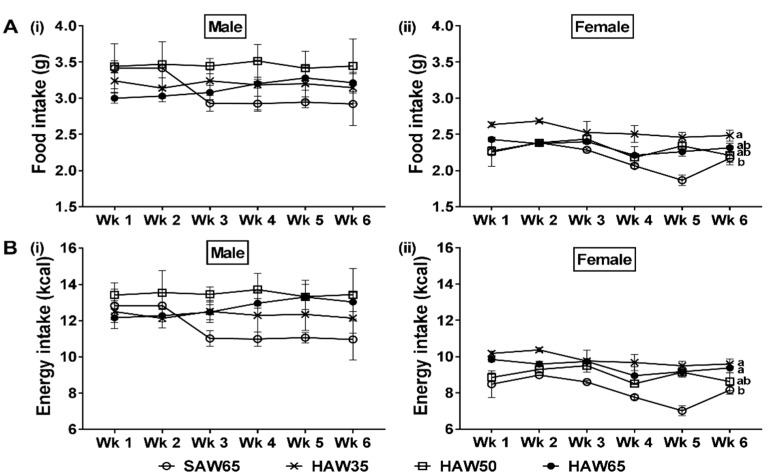
Weekly changes of (**A**) food intake and (**B**) energy intake in (i) male and (ii) female mice fed diets containing 65% standard amylose wheat (SAW65), 30% SAW and 35% high amylose wheat (HAW35), 15% SAW and 50% HAW (HAW50) or 65% HAW (HAW65) for six weeks. Data are means ± SEM (*n* = 8 mice/group). Values with different superscripts indicate significant difference (*p* < 0.05) by two-way ANOVA followed by Tukey’s post hoc test.

In males, there were no differences between diet groups in either food intake or energy intake across the first six weeks of the feeding period (Figure 1(Ai,Bi)). In females, however, the HAW35 group had higher food intake (*p* = 0.02; Figure 1(Aii)) and energy intake (*p* < 0.01; Figure 1(Bii)), across the six-week feeding period, compared to the SAW65 group. The energy intake of the HAW65 females was higher than the SAW65 group (*p* = 0.02) during this time, but food intake was not different between these two groups.

### 3.2. Energy Expenditure, RQ, Water Intake and Ambulatory Activity

**Figure 2:**
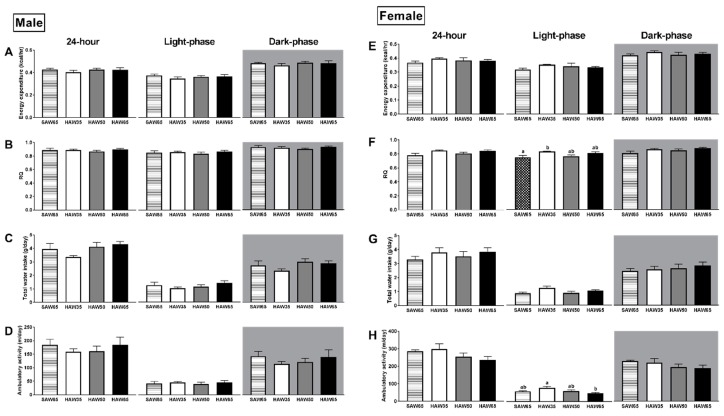
Metabolic parameters of mice fed diets containing 65% standard amylose wheat (SAW65), 30% SAW and 35% high amylose wheat (HAW35), 15% SAW and 50% HAW (HAW50) or 65% HAW (HAW65) in weeks 7 and 8. Twenty-four-hour, light- and dark-phase average energy expenditure (**A**: male; **E**: female); average RQ (**B**: male; **F**: female); total water intake (**C**: male; **G**: female); and total ambulatory activity of (**D**) male and (**H**) female mice are shown. Data are means ± SEM (*n* = 7 or 8 mice/group). Values with different superscripts indicate significant difference (*p* < 0.05) by one-way ANOVA followed by Tukey’s post hoc test.

Overall, both males and females exhibited higher energy expenditure, RQ, water intake and ambulatory activity in the dark phase compared to the light phase. In males, there were no differences in energy expenditure (Figure 2A), RQ (Figure 2B), water intake (Figure 2C) or ambulatory activity (Figure 2D) between diet groups. In females, there were no differences in energy expenditure (Figure 2E) or water intake (Figure 2G) between diet groups. However, females consuming the HAW35 diet exhibited a higher RQ compared to the SAW65 group (*p* = 0.03; Figure 2F) and higher ambulatory activity compared to the HAW65 group (*p* = 0.02; Figure 2H), during the light phase only.

### 3.3. Body Weight, Abdominal Circumference, Nose–Anus Length, Relative Fat Mass and Relative Organ Weights

**Figure 3:**
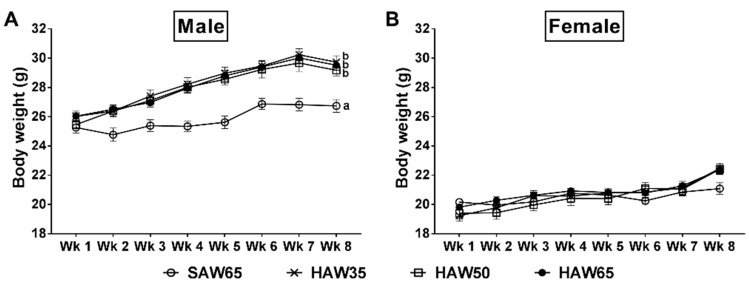
Weekly changes of body weight in (**A**) male and (**B**) female mice fed diets containing 65% standard amylose wheat (SAW65), 30% SAW and 35% high amylose wheat (HAW35), 15% SAW and 50% HAW (HAW50) or 65% HAW (HAW65) for eight weeks. Data are means ± SEM (*n* = 8 mice/group). Values with different superscripts indicate significant difference (*p* < 0.05) by two-way ANOVA followed by Tukey’s post hoc test.

Table 3 shows BW, abdominal circumference, nose–anus length, relative fat mass and relative organ weights of male and female mice after the eight-week feeding period. In males, mice consuming a diet containing any level of HAW were heavier than those that consumed the SAW65 diet during (*p* < 0.05; Figure 3A) and at the end (*p* < 0.05) of the eight-week feeding period. The BW gain across the eight-week feeding period was also higher in all groups of male mice consuming any level of HAW compared to the SAW65 males (*p* < 0.05). Males consuming the diet which contained the highest level of HAW (HAW65) had a lower abdominal circumference compared to the HAW35 males after the eight weeks (*p* = 0.04), but nose–anus length was not different between groups. Relative total fat mass (24.48 ± 2.50 vs. 40.78 ± 3.00 mg/g BW; *p* = 0.01) and relative gonadal fat mass (9.93 ± 1.35 vs. 18.51 ± 1.59 mg/g BW; *p* < 0.01) were lower in the HAW65 males compared to the HAW35 males. The HAW35 males did, however, have a higher relative gonadal fat mass (18.51 ± 1.59 vs. 11.10 ± 2.00 mg/g BW; *p* = 0.02) compared to the SAW65 males. There were no differences in the relative weights of other fat mass depots (retroperitoneal, mesentery and interscapular) between diet groups. Similarly, no differences were observed in the relative weights of kidneys, liver, pancreas or spleen between groups (Table 3).

In females, there were no differences between diet groups in the BW either during (Figure 3B) or at the end (Table 3) of the eight-week feeding period. However, the amount of BW gained across the eight-week feeding period was higher in the HAW35 females compared to the SAW65 group (*p* = 0.03). There were no differences between diet groups in abdominal circumference, nose–anus length or relative weights of total fat, individual fat depots, kidneys, liver, pancreas or spleen at the end of the feeding period (Table 3).

### 3.4. Plasma Hormone and Metabolite Concentrations

In males, there were no differences between diet groups in the fasting blood glucose concentrations or fasting plasma concentrations of total cholesterol, triglycerides or non-esterified fatty acids at the end of the eight-week feeding period (Table 4). However, the HAW35 males (0.48 ± 0.12 ng/mL) had higher fasting leptin concentrations compared to both HAW65 males (0.09 ± 0.03 ng/mL; *p* < 0.01) and SAW65 males (0.16 ± 0.06 ng/mL; *p* = 0.02). In females, no differences were observed between diet groups in the fasting blood glucose concentrations or fasting plasma concentrations of total cholesterol, triglycerides, non-esterified fatty acids or leptin at the end of the experiment (Table 4).

### 3.5. Markers of Reproductive Function

**Figure 4:**
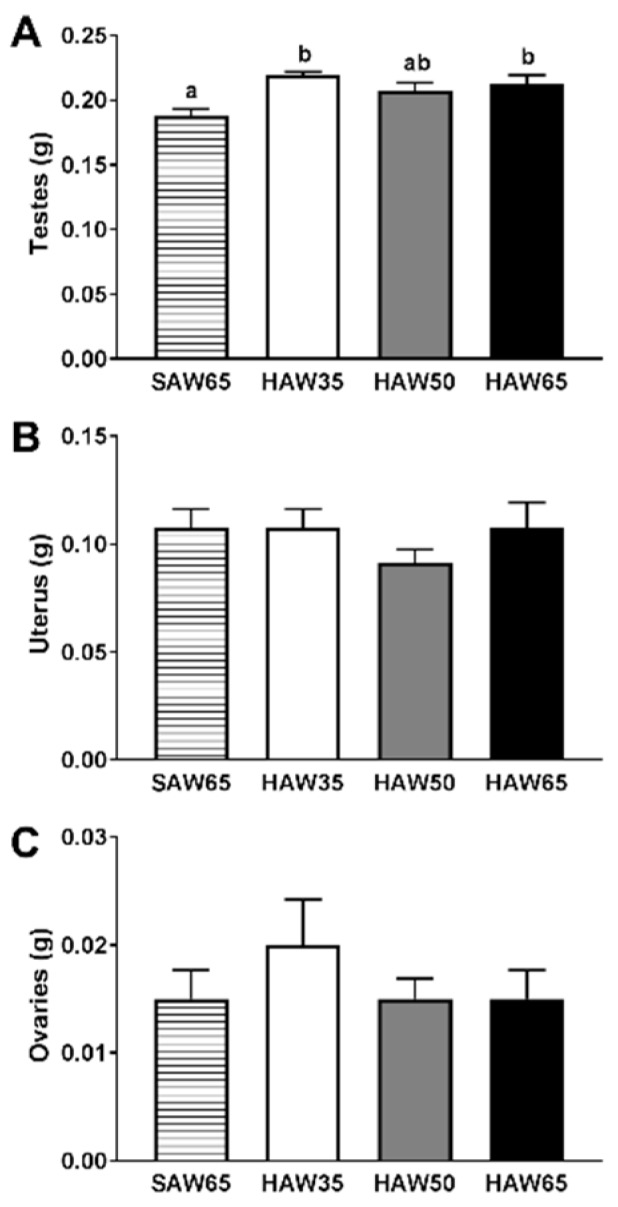
Weights of (**A**) testes or (**B**) uterus and (**C**) ovaries in male and female mice fed diets containing 65% standard amylose wheat (SAW65), 30% SAW and 35% high amylose wheat (HAW35), 15% SAW and 50% HAW (HAW50) or 65% HAW (HAW65) at the end of the eight-week feeding period. Data are means ± SEM (*n* = 8 mice/group). Values with different superscripts indicate significant difference (*p* < 0.05) by one-way ANOVA followed by Tukey’s post hoc test.

**Figure 5:**
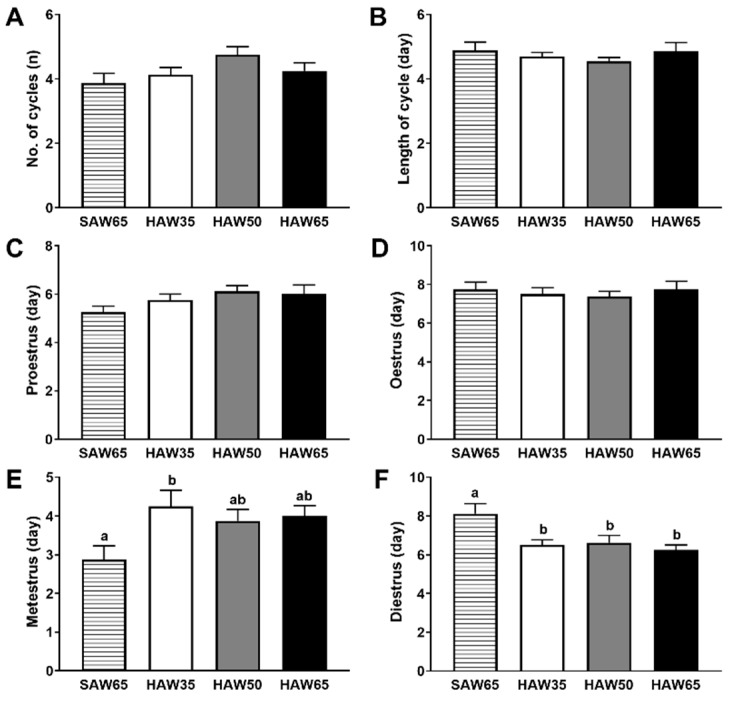
Average (**A**) number and (**B**) length in days of oestrus cycles and total number of days in (**C**) proestrus, (**D**) oestrus, (**E**) metestrus and (**F**) diestrus in female mice fed diets containing 65% standard amylose wheat (SAW65), 30% SAW and 35% high amylose wheat (HAW35), 15% SAW and 50% HAW (HAW50) or 65% HAW (HAW65) over 24 days from weeks 4 to 7. Data are means ± SEM (*n* = 8 mice/group). Values with different superscripts indicate significant difference (*p* < 0.05) by one-way ANOVA followed by Tukey’s post hoc test.

In males, testes weights at the eight-week time point were higher in both HAW35 (*p* < 0.01) and HAW65 (*p* = 0.02) groups, and tended to be higher in the HAW50 group (*p* = 0.07) compared to the SAW65 group (Figure 4A). In females, there were no differences between diet groups in the weights of the uterus (Figure 4B) or ovaries (Figure 4C) at eight weeks. There were no differences in the average number (Figure 5A) or length (Figure 5B) of the oestrus cycles between diet groups. However, females that were fed on diets containing any level of HAW spent a lower proportion of the assessment period in diestrus (*p* < 0.05; Figure 5F) and tended to spend more time in metestrus compared to the SAW65 group (Figure 5E). There were no differences between diet groups in the proportion of time mice spent in either proestrus (Figure 5C) or oestrus (Figure 5D).

## 4. Discussion

The results of the current study suggest that replacing standard wheat with levels of HAW between 35% and 65% of total diet weight did not produce any substantive effects on fasting blood glucose or plasma lipid profile in either male or female lean mice. There were, however, a number of effects of increased HAW consumption on measures of nutrient metabolism (RQ), growth and body fat mass, which appeared to be dependent on both the sex of the animals and the level of HAW in the diet. We also observed interesting effects of the HAW diets on reproductive organ weights in males and oestrus cyclicity in females, which raises the possibility of effects on reproductive function which warrant further investigation.

The higher BW observed at the end of the eight-week feeding period in male mice fed on any of the HAW diets is consistent with a previous study in male Wistar rats fed a diet containing 20% HAW [22]. In both the previous [22] and current studies, the increase in BW occurred in the absence of any differences in feed intake, suggesting that it was not a result of any appetite-promoting effects of the HAW diet. Moreover, in the previous study [22], the higher BW of rats fed a 20% HAW diet was attributed to an increase in muscle and bone mineral mass. It is possible that the increased BW in male mice consuming the HAW diets in this study was due to increased accumulation of muscle, and potentially bone mass, however, further studies are required to confirm this directly. Unlike BW, an intriguing finding of this study was that the effects of the HAW diets on fat mass appeared to be dependent on the level of HAW included in the diet, and that fat mass relative to BW was actually higher in the mice consuming the lowest level of HAW (HAW35) compared to those consuming the highest level (65%). This effect was particularly pronounced for abdominal fat pads, and raises the possibility that higher levels of HAW consumption are required to reduce abdominal adiposity. This is important, given that increased abdominal adiposity is a major risk factor for cardiometabolic disease and mortality [23]. If the positive impact of HAW on fat deposition in males is translated to humans, this has the potential to reduce the risk of cardiometabolic disease, particularly in men who have a greater tendency to accumulate fat around the abdomen than women.

In line with previous studies investigating the effects of a range of dietary interventions [24,25,26], we observed that the male and female mice exhibited quite different responses to the consumption of increased amounts of HAW in the diet in relation to effects on food intake, RQ, BW and fat mass. In contrast to the findings of male mice, female mice consuming the lowest level of HAW diet (HAW35) exhibited higher intakes of food and energy compared to those consuming the SAW diet, and this was also associated with increased BW gain. However, fat mass as a proportion of BW tended to be lower in female mice consuming any level of HAW diet compared to the SAW65 group, suggesting that the increase in BW gain was not due to excess fat accumulation. This may suggest an effect on muscle and/or bone mass of HAW. The higher intakes of food and energy in HAW35 female group were correlated with an increase in RQ during the light phase compared to the SAW65 group, suggesting preferential utilisation of carbohydrate as a fuel source during this period. The increased RQ during the light phase may be due to either an increase in food intake during the light phase or the slower transition and digestion of the HAW diet and therefore delayed provision of carbohydrates to the light phase. The method used for food intake measurement in this study prevented analysis of changes in food intake patterns and, therefore, further studies are required to determine the exact reason for the increased RQ during the light phase, and the impact of this on energy utilization and body fat mass in the longer term. If the observed effect on RQ in females is translated to humans, it would be expected to be associated with a greater utilization of carbohydrate as an energy source. This, in turn, would provide a more consistent glucose supply, but may also limit the utilisation of endogenous fat stores, which could potentially lead to increases in body fat in the longer term when consuming a high-carbohydrate diet. The differences in response to diets between males and females were likely due to a number of factors, including sex-specific differences in metabolic control, body fat distribution, sex hormones and taste preferences [27,28]. Nonetheless, the current study does clearly demonstrate that the response to the same level of HAW in the diet has different effects on the measured parameters in males and females, and that it is not appropriate to extrapolate results obtained in male mice to their female counterparts.

Dietary fibre plays an important role in maintaining good health and is associated with improvement of glycaemic response and plasma lipid profile [29,30]. However, consistent with previous findings [22], the HAW diets had no effects on fasting blood glucose concentrations or plasma lipid profiles despite the higher dietary fibre content in the HAW diets compared to the SAW diet. One possible explanation is that both SAW and HAW contain similar types of soluble fibres including arabinoxylans, β-glucans and inulin type fructans, which have previously been shown to lower plasma cholesterol and improve glucose and lipid metabolism [31,32]. It is important to note that the mice in this study were lean and metabolically healthy, and this may have limited the potential to produce further improvements in glucose/lipid control. It is possible that more pronounced differences would be observed if mice were metabolically compromised (e.g., diabetic or hyperlipidemic); however, this requires further investigation.

Reproductive health is increasingly recognised to be tightly linked to metabolic health [18], and to be strongly influenced by diet and lifestyle factors [33]. This prompted us to investigate the potential effects of the HAW diet on markers of reproductive function. Clinical and experimental animal studies indicate that testicular volume is positively associated with sperm production and sperm motility [34,35,36,37], both markers of male reproductive health, and clinical studies show that this is also related to circulating sex hormone concentrations [36,37]. This suggests that the higher testicular weights in mice consuming diets containing HAW compared to those consuming the SAW65 diet may be associated with improved sperm characteristics and sex hormone concentrations. Female mice consuming any level of the HAW diets showed an altered pattern of vaginal cytology parameters, which suggested a shift in the oestrus cycle towards a longer period in metestrus and a shorter period in diestrus compared to those consuming the SAW65 diet. Prolonged diestrus is a commonly observed pattern in rodent models of disordered fertility [38], raising the possibility that the HAW diet had a positive impact on reproductive health in females. The changes observed may also be indicative of effects on sex hormone concentrations [39] or immune determinants of reproductive function [40], which were not measured in the current study. Immune cell populations residing in female reproductive tissues change across the oestrus cycle in response to ovarian hormone levels, with the largest populations present in tissues and sequestered into the vaginal epithelium at oestrus and metestrus stages when oestrogen levels are high [41,42]. Consequently, the longer metestrus phase may reflect altered immune cell populations or function in the reproductive tract, and potentially these are linked with receptivity to pregnancy. Further studies that include more direct and robust measures of reproductive function in males and females will be important to enable us to draw definitive conclusions as to the potential benefits of HAW diets on reproductive performance. The effects of HAW consumption on reproductive parameters observed in this study also raise the possibility that replacing SAW with HAW in human staple foods may provide reproductive health benefits, although it is clear that further studies are required to address this directly.

## 5. Conclusions

This study demonstrates that replacement of SAW flour with increasing levels of HAW in diets of healthy, lean male and female mice was associated with sex-specific effects on growth, fat mass and nutrient metabolism, but in the absence of any effects on fasting glucose or blood lipid concentrations. The level of HAW also appeared to be important, with different effects on fat deposition, nutrient metabolism and growth observed between the diets containing the lowest and highest levels of HAW. The reason for this is not clear, but these results suggest that considering the level of HAW included in experimental diets is important when comparing studies evaluating effects of this wheat type. Further research is required to determine the metabolic effects of a HAW diet in metabolically compromised models. The differences in reproductive parameters seen in HAW-compared to SAW-fed male and female mice also require further investigation.

## Figures and Tables

**Table 1:** Composition of experimental diets.

Ingredient (g)	SAW65	HAW35	HAW50	HAW65
Standard amylose wheat	650.00	300.00	150.00	0.00
High amylose wheat	0.00	350.00	500.00	650.00
Maltodextrin	42.97	42.97	42.97	42.97
Sucrose	27.72	27.72	27.72	27.72
Casein	140.00	140.00	140.00	140.00
L-cystine	1.80	1.80	1.80	1.80
Soybean oil	40.00	40.00	40.00	40.00
Cellulose	50.00	50.00	50.00	50.00
Mineral mix, AIN-93M-MX	35.00	35.00	35.00	35.00
Vitamin mix, AIN-93-VX	10.00	10.00	10.00	10.00
Choline bitartrate	2.50	2.50	2.50	2.50
TBHQ, antioxidant	0.008	0.008	0.008	0.008
Total	1000	1000	1000	1000

SAW, standard amylose wheat; HAW, high amylose wheat.

**Table 2:** Nutrient analysis of experimental diets ^1^.

	SAW65	HAW35	HAW50	HAW65
Energy (kcal/g)	3.76	3.86	3.90	4.05
Moisture (g/100 g)	10.4	8.2	8.1	5.9
Fat (Mojonnier extraction; g/100 g)	5.5	6.4	6.9	7.6
Protein (N × 6.26; g/100 g)	20.9	21.7	21.7	22.5
Ash (g/100 g)	3.1	3.3	3.8	3.5
Carbohydrates (by difference; g/100 g)	60	60	60	61
Calcium (mg/kg)	5000	5000	5300	5300
Iron (mg/kg)	80	72	82	83
Total dietary fibre (%)	13.5	16.2	17.3	18.5
Insoluble dietary fibre (%)	10.4	12.1	12.9	13.6
Soluble dietary fibre (by difference; %)	3.0	4.0	4.5	4.9
Amylopectin (%)	36.2	31.5	29.5	27.5
Amylose (%)	4.5	4.2	3.4	<4.0
Total Starch (%)	40.7	33.6	30.5	27.5
Resistant Starch (%)	1.2	1.6	1.7	1.9
Rapid Digestibility (%)	12.7	11.9	11.6	11.3
Slow Digestibility (%)	32.8	27.5	25.3	23.0

SAW, standard amylose wheat; HAW, high amylose wheat. ^1^ The fibre, starch and digestibility measures of HAW35 and HAW50 diets were calculated based on the proportions of SAW and HAW in the diet.

**Table 3:** Body weight, abdominal circumference, nose–anus length, relative fat mass and relative organ weights of male and female mice at the end of the eight-week feeding period.

	SAW65	HAW35	HAW50	HAW65
**Male**				
End point BW ^1^ (g)	22.64 ± 0.41 ^a^	25.96 ± 0.26 ^b^	25.01 ± 0.34 ^b^	24.56 ± 0.43 ^b^
Weight gain ^2^ (g)	1.89 ± 0.21 ^a^	3.71 ± 0.30 ^b^	3.71 ± 0.29 ^b^	3.48 ± 0.33 ^b^
Abdominal circumference (cm)	7.04 ± 0.10 ^ab^	7.28 ± 0.11 ^a^	6.79 ± 0.14 ^ab^	6.76 ± 0.16 ^b^
Nose–anus length (cm)	9.16 ± 0.06	9.24 ± 0.10	9.10 ± 0.04	9.08 ± 0.06
Total fat mass (mg/g BW)	28.58 ± 4.95 ^ab^	40.78 ± 3.00 ^a^	32.72 ± 2.84 ^ab^	24.48 ± 2.50 ^b^
Gonadal (mg/g BW)	11.10 ± 2.00 ^a^	18.51 ± 1.59 ^b^	14.42 ± 1.55 ^ab^	9.93 ± 1.35 ^a^
Retroperitoneal (mg/g BW)	4.15 ± 0.74	6.55 ± 1.19	4.59 ± 0.94	3.25 ± 0.57
Mesentery (mg/g BW)	8.75 ± 1.64	10.29 ± 0.55	8.02 ± 0.55	7.42 ± 0.58
Interscapular (mg/g BW)	4.58 ± 0.79	5.43 ± 0.46	5.69 ± 0.43	3.89 ± 0.47
Kidneys (mg/g BW)	11.98 ± 0.22	12.81 ± 0.47	12.49 ± 0.22	13.09 ± 0.44
Liver (mg/g BW)	40.40 ± 0.54	38.74 ± 1.80	43.39 ± 0.92	42.42 ± 2.06
Pancreas (mg/g BW)	4.41 ± 0.29	4.53 ± 0.16	3.92 ± 0.45	3.92 ± 0.17
Spleen (mg/g BW)	2.36 ± 0.11	2.60 ± 0.13	2.40 ± 0.10	2.44 ± 0.14
**Female**				
End point BW ^1^ (g)	17.04 ± 0.20	18.21 ± 0.38	18.05 ± 0.40	18.01 ± 0.23
Weight gain ^2^ (g)	1.37 ± 0.43 ^a^	3.23 ± 0.31 ^b^	3.00 ± 0.59 ^ab^	2.56 ± 0.30 ^ab^
Abdominal circumference (cm)	6.56 ± 0.06	6.59 ± 0.10	6.63 ± 0.06	6.45 ± 0.08
Nose–anus length (cm)	8.55 ± 0.06	8.80 ± 0.06	8.70 ± 0.08	8.60 ± 0.09
Total fat mass (mg/g BW)	27.58 ± 0.99	23.89 ± 2.64	23.39 ± 1.84	23.33 ± 1.04
Gonadal (mg/g BW)	10.82 ± 0.74	8.43 ± 1.22	9.02 ± 1.05	8.52 ± 0.64
Retroperitoneal (mg/g BW)	2.43 ± 0.24	2.40 ± 0.38	2.85 ± 0.32	2.15 ± 0.16
Mesentery (mg/g BW)	8.43 ± 1.28	7.38 ± 0.82	6.89 ± 0.44	8.40 ± 0.52
Interscapular (mg/g BW)	5.89 ± 0.43	5.68 ± 0.63	4.63 ± 0.52	4.26 ± 0.32
Kidneys (mg/g BW)	13.23 ± 0.30	14.26 ± 0.38	13.40 ± 0.48	13.15 ± 0.96
Liver (mg/g BW)	42.61 ± 0.49	40.64 ± 2.04	43.70 ± 1.02	45.08 ± 0.93
Pancreas (mg/g BW)	4.62 ± 0.30	4.31 ± 0.34	4.22 ± 0.42	4.53 ± 0.28
Spleen (mg/g BW)	3.09 ± 0.16	3.93 ± 0.58	3.22 ± 0.39	3.06 ± 0.15

Mice fed diets containing 65% standard amylose wheat (SAW65), 30% SAW and 35% high amylose wheat (HAW35), 15% SAW and 50% HAW (HAW50) or 65% HAW (HAW65). ^1^ Measured before blood and tissue collection at fasted state. ^2^ Difference between the body weights of week 8 and pre-intervention values. Data are means ± SEM (*n* = 8 mice/group). Values with different superscripts in a row indicate significant difference (*p* < 0.05) by one-way ANOVA followed by Tukey’s post hoc test.

**Table 4:** Biochemical analyses of blood/plasma from male and female mice at the end of the eight-week feeding period.

Parameter	SAW65	HAW35	HAW50	HAW65
Male				
Fasting blood glucose (mmol/L)	7.59 ± 0.48	8.15 ± 0.66	8.14 ± 0.69	8.20 ± 0.63
Total cholesterol (mmol/L)	2.54 ± 0.07	2.66 ± 0.23	2.50 ± 0.06	2.35 ± 0.21
Triglycerides (mmol/L)	0.76 ± 0.04	0.85 ± 0.06	0.94 ± 0.06	0.87 ± 0.06
Non-esterified fatty acids (mmol/L)	0.94 ± 0.08	0.84 ± 0.04	0.91 ± 0.08	1.00 ± 0.07
Leptin (ng/mL)	0.16 ± 0.06 ^a^	0.48 ± 0.12 ^b^	0.21 ± 0.04 ^ab^	0.09 ± 0.03 ^a^
Female				
Fasting blood glucose	6.58 ± 0.45	7.43 ± 0.47	7.30 ± 0.28	7.11 ± 0.18
Total cholesterol (mmol/L)	1.70 ± 0.03	1.71 ± 0.11	1.61 ± 0.10	1.72 ± 0.05
Triglycerides (mmol/L)	0.74 ± 0.03	0.70 ± 0.03	0.70 ± 0.03	0.71 ± 0.04
Non-esterified fatty acids (mmol/L)	0.81 ± 0.03	0.82 ± 0.06	0.88 ± 0.02	0.80 ± 0.05
Leptin (ng/mL)	0.15 ± 0.03	0.10 ± 0.02	0.14 ± 0.04	0.18 ± 0.03

Mice fed diets containing 65% standard amylose wheat (SAW65), 30% SAW and 35% high amylose wheat (HAW35), 15% SAW and 50% HAW (HAW50) or 65% HAW (HAW65). Data are means ± SEM (*n* = 8 mice/group). Values with different superscripts in a row indicate significant difference (*p* < 0.05) by one-way ANOVA followed by Tukey’s post hoc test.

## Supplementary Materials

The following is available online at https://www.mdpi.com/2072-6643/12/1/61/s1, Table S1: Nutrient analysis of high amylose wheat used in this study.
