# Supplementary material for: Sexually Dimorphic Response of Increasing Dietary Intake of High Amylose Wheat on Metabolic and Reproductive Outcomes in Male and Female Mice
Source: Nutrients. 2019 Dec 25;12(1):61. doi: 10.3390/nu12010061 (PMC7019933; doi:10.3390/nu12010061)
Supplement: Supplementary file 1 [file nutrients-12-00061-s001.pdf]

Table S1. Nutrient analysis of high amylose wheat used in this study.

**Test**	**%**
Moisture	8.80
Total starch	33.30
Amylose	15.46
Amylopectin	17.80
Total dietary fibre	24.90
Insoluble dietary fibre	21.80
Soluble dietary fibre (by difference)	3.10
Resistant starch	4.50
Beta-glucan	1.80
Sugars	3.20
Inulin	7.40
